# From Autonomy Support and Grit to Satisfaction With Life Through Self-Determined Motivation and Group Cohesion in Higher Education

**DOI:** 10.3389/fpsyg.2020.579492

**Published:** 2021-01-08

**Authors:** José Eduardo Lozano-Jiménez, Elisa Huéscar, Juan Antonio Moreno-Murcia

**Affiliations:** ^1^Faculty of Human and Social Sciences, Universidad de la Costa, Barranquilla, Colombia; ^2^Department of Health Sciences, Miguel Hernández University, Elche, Spain; ^3^Department of Sport Sciences, Sport Research Centre, Miguel Hernández University, Elche, Spain

**Keywords:** motivation, cohesion, university students, teaching style, autonomy support

## Abstract

Using the Self-Determination Theory as a framework, this study tests the predictive capacity of the teacher’s interpersonal style of autonomy support at a higher education institution, and the grit on the satisfaction of basic psychological needs, intrinsic motivation, group cohesion, and life satisfaction in university students. A sample composed of 489 Colombian university students (381 women and 108 men), aged between 18 and 41 years (*M* = 21.93; *DT* = 3.58), was used; they filled in the questionnaires that measured the variables of interest. After the analysis of structural equations, the results showed that the perception of teaching style of autonomy support and the grit positively predicted the basic psychological needs and these predicted the intrinsic motivation, which in turn predicted group cohesion and satisfaction with life. The model describes the possible importance of promoting the teacher’s interpersonal style of autonomy support within the university setting in the search for satisfaction with life along with the active role of the student through the mediation of the satisfaction of basic psychological needs, increased quality motivation, and high group cohesion.

## Introduction

Motivational aspects are considered important promoters of success in the educational setting (McLachlan and Hagger, 2010). The Self-Determination Theory (SDT; Ryan and Deci, 2017) has indicated that social contexts are key to generate greater well-being (Ryan and Deci, 2000). But at the same time, the SDT also explains that some personal factors play a determining role in this process along with contextual factors. In this sense, the research so far has indicated that students with higher grit scores (consistency and perseverance) tend to work more persistently (Seong-Lee and Chen-Hsieh, 2019) and achieve greater psychological well-being (Cortez et al., 2019). Furthermore, numerous adaptive outcomes such as well-being and academic success are also associated with group cohesion through building positive bonds between students (Marmarosh and Markin, 2007; Thornton et al., 2019). In this teaching scenario, the teaching role can become a powerful social trigger that promotes adaptive outcomes through certain interpersonal styles (Leenknecht et al., 2017) that, added to the existence of a grit in a student (high in grit), can enhance student motivation in dynamics that promote group cohesion as well as their perceived well-being (Bronson, 2016). Considering all of this, we attempt to go deeper into testing a new motivational model that allows understanding the relationship between these variables in higher education students.

### Social and Personal Triggers

The way in which teachers interact with their students is a central component in the SDT; through their behavior, the teacher can promote positive and adaptive behaviors in their students. Autonomy support versus the controlling style is the teaching style that has demonstrated a positive impact in the academic context.

Self-Determination Theory, centered on the bright view of motivation, proposes that the style of autonomy support is a predictor of the satisfaction of the basic psychological needs (BPN) of autonomy, competence, and relationship with others (Ryan and Deci, 2000). The latter is related to the provision of social resources by people’s networks, which is in line with what is suggested by Rocchi et al. (2017). In turn, BPNs are predictors of self-determined motivation. Specifically, autonomy support is situated as a central social trigger for the development of self-determined motivation in students (Zamzami and Corinne, 2019) and as a key element for greater academic achievement and permanence (Leenknecht et al., 2017), insofar as it seeks to enhance not only autonomy and competence but also social support, understood as a relationship with others, in recognition of the key role that others play for the experience of people (Stroet et al., 2013). In the opposite sense, a controlling style that does not enhance students’ BPN, including frustration in relationships, is directly related to an increase in amotivation (Martinek et al., 2020). Incorporating activities in the classroom based on providing autonomy support can lead to a better student perception of classroom instruction, giving the teacher a higher grade, improving both their motivation and learning (Griffin, 2016), and fostering greater commitment with their studies and their performance (Bronson, 2016).

The literature has highlighted the role of social triggers in satisfying basic psychological needs to promote intrinsic motivation, which in turn would be related to different effects (Haerens et al., 2015). Just as the social trigger that the teacher represents can promote quality motivation, the individual characteristics of the students also participate, and these may promote or hinder said relationship. In this sense, recent works highlight the value of taking into account non-cognitive traits in the educational setting; these non-cognitive traits, as the name indicates, do not have to do with the intellect but rather with temperamental, attitudinal, and motivational characteristics of the student (Fomunyam and Mnisi, 2017). Thus, the grit as a personal factor that the students display interacts with the interpersonal teaching style and must be taken into account. Grit is defined as consistency and perseverance toward long-term goals and describes a sustained commitment to complete a task that involves effort despite failures, setbacks, and adversities (Duckworth et al., 2007), Therefore, it shows a strong relationship with the student’s capacity for self-control (González et al., 2019). From recent literature, we know that through grit, students can enhance their own motivation, achievement, and well-being (Cortez et al., 2019; Seong-Lee and Chen-Hsieh, 2019). According to Akbağ and Ümmet (2017), grit and the satisfaction of basic psychological needs, as well as gender, are significant predictors of subjective well-being in young adults, having a positive and statistically significant relationship with each other. Specifically in a study among university students, Scherer et al. (2017) stressed the need to structure programs that develop the dispositional factors related to grit for academic success and retention. Miller-Matero et al. (2018) concluded that grit is related to academic performance, in that students who show high levels of perseverance are more likely to perform better. Borae and Kim (2017) concluded that the satisfaction of basic psychological needs is associated with grit and in turn with subjective well-being. In the same sense, Isenberg et al. (2020) conclude that grit is positively associated with personal well-being and with aspects of personality such as relationship building and empathy regarding the sense of group.

### Satisfaction of Basic Psychological Needs

The SDT (Ryan and Deci, 2017) proposes as a key aspect that people have a natural desire to experience a sense of choice and psychological freedom regarding their thinking and actions. In other words, people have a tendency toward autonomous motivation and self-determination. This involves both intrinsic motivation and integrated regulation. The first, always autonomous, allows the development of an activity in an optimal and challenging way, from an internal locus of causality, and that is invigorated by basic psychological needs, without the need for external incentives. Extrinsic motivation involves developing an activity motivated by a reward or the avoidance of punishment. However, it can become autonomous, through internalization and integration processes, which tend to occur in diverse social settings such as home and school, among others (Deci, 2004). Although, finally, motivation rests on a continuum of processes that go from amotivation, to intrinsic motivation, through introjection, to integrated motivation (Ryan and Deci, 2020), various studies support the idea that intrinsic motivation it is highly beneficial in formal education (Taylor et al., 2014; Froiland and Worrell, 2016). Although so is integrated regulation, intrinsic motivation is a natural and inherent component of the human condition, which moves it toward action for the sake of its own psychological growth. Its mere existence allows it to be strengthened, as it is not an automatic process, and the need to seek scenarios that consolidate it is recognized, such as the condition of autonomy support by teachers. Although integrated regulation also has effects on individual well-being, and it is usual for an action to be driven by both intrinsic and integrated regulation, the latter represents an extensive route for its emergence and maintenance, directed from externality to integration. According to various studies (Khalaila, 2014; Negovan et al., 2015; Griffin, 2016; Weidinger et al., 2016), focusing on intrinsic motivation allows starting from the natural tendency and enhancing it in a shorter way and, according to the SDT, with conditions focused on the satisfaction of the BPN (Ryan and Deci, 2020). When people have the basic psychological needs of autonomy, competence and relationship with others satisfied, self-determined motivation is promoted and, therefore, a large number of positive results are achieved (Orsini et al., 2018). In the educational context, intrinsic motivation is a key factor in the learning process (Depasque and Tricomi, 2015; Tahrekhani and Sadeghian, 2015). In particular, regarding autonomy, the action of choosing voluntarily, in a self-determined way, promotes intrinsic motivation and greater effort in tasks (Meng and Ma, 2015). Although various studies have approached BPN in a discriminated way, others (Orsini et al., 2018; Kingsford-Smith and Evans, 2019; Li et al., 2019; Tavernier et al., 2019) have done it jointly, showing unanimity regarding positive adaptive results. To nurture students’ BPNs, teachers as social triggers must adopt an interactional style that supports autonomy, which implies instructing in the possibility of choice, building learning based on the design of a clearly defined structure, and promoting relationships between students (Soenens et al., 2018). When teachers support autonomy, students have more opportunities to take initiative and play a leadership role (Vermote et al., 2020), as they catalyze greater intrinsic motivation, curiosity, and desire for challenge (Ryan and Deci, 2000), developing a more self-determined motivation and achieving the satisfaction of their basic psychological needs (Frielink et al., 2018).

### Group Cohesion

Unlike the concept of relationship with others, which refers to the need for people to get involved with others and feel part of a collective through links (Ryan and Deci, 2000), group cohesion focuses on the individual sense of belonging to a group along with the moral feelings associated with the other members of the group (Bollen and Hoyle, 1990). Specifically, well-being and academic success in college students are associated with bonding and group cohesion (Marmarosh and Markin, 2007). In the same sense, Bravo et al. (2018) point out that it is key to incorporate teamwork tasks for collaborative learning in the teaching practices at the higher education level; this style of interrelation and direction in the classroom can increase individual achievement, more so than purely individual or competitive learning. In this direction Slavin (2014) analyzes the role of social cohesion in collaborative learning, as one of the four theoretical alternatives to study performance, and points out the importance of team building and the quality of group interaction for such end.

### Satisfaction With Life

Life satisfaction, understood as a cognitive component of subjective well-being, refers to the global evaluation that the person makes of their satisfaction with life (Diener, 2000). Its relationship with autonomy support in university students has been previously explored in different settings. Kim et al. (2019) found that the interaction with many other heterogeneous people through online social networks is related to both satisfaction with life on campus and with the perception of self-efficacy and personal well-being. In the same sense, Pang (2018) concludes that the intensity of the use of microblogs is positively associated with the maintenance of friendship and satisfaction with the life of the students, who by revealing their thoughts and emotions with other online users sustain friendships and achieve greater satisfaction with life. Although Moreno-Murcia et al. (2020) in a cross-cultural study concluded that perceived autonomy support is positively associated with the satisfaction of psychological needs, intrinsic motivation, and group cohesion, which suggests the promotion of positive social relationships among university students, no investigations have been found in which, added to these, grit is included as a key trigger in this process, which represents a considerable contribution of the present study.

Initial studies already indicate the importance of consolidating a solid motivational model based on SDT, to promote well-being in university students (Martín-Albo et al., 2009). Autonomy promotion strategies ensure a favorable environment for learning (Bronson, 2016). In this same sense, Leenknecht et al. (2017) state that teachers who support autonomy promote their students’ intrinsic motivation and achievement. This study focuses on testing the predictive capacity of the teacher’s interpersonal style of autonomy support as well as the subjective consistency and perseverance on the satisfaction of basic psychological needs, intrinsic motivation, group cohesion, and satisfaction with life, in university students. Therefore, it is expected that the interpersonal style of autonomy support and grit positively explain the satisfaction of basic psychological needs, and these would then explain the intrinsic motivation that is expected would lead to greater satisfaction with life, mediated by group cohesion.

## Materials and Methods

### Participants

The sample was made up of 489 Colombian university students (381 women and 108 men) from different levels of the Psychology Program of the Universidad de la Costa de Barranquilla (21 in 2nd semester; 47 in 3rd semester; 153 in 5th semester; 47 in 6th semester; 66 in 7th semester; 99 in 8th semester; 56 in 9th semester), with ages between 18 and 41 years (*M* = 21.93; *DT* = 3.58), and, in general, from socioeconomic strata 1 and 2 (out of 5), characterized by levels of skill development below the national average. They were selected through an intentional sampling, considering the availability of teachers at the time of administration of the instruments. Those in the first semester were not included because they were just beginning neither their training, nor those in the tenth semester because they were outside the university and advancing their professional practices.

### Measurements

#### Autonomy Support

To measure the interpersonal style of autonomy support that the Higher Education student perceives of their teacher, the Moreno-Murcia et al. (2019) *Autonomy Support Scale* (*EAA*) was used. It consists of 12 items (e.g., “Provide explanations that help us understand the personal usefulness of carrying out this activity”) and the scale begins with an introductory heading such as: “My teacher in class …”. This is valued on a Likert scale from 1 (*Strongly disagree*) to 5 (*Strongly agree*). The results of confirmatory factor analysis were satisfactory: *χ*^2^ = 3.87; *p* = 0.56; *χ*^2^/d.f. = 1.23; CFI = 0.99; NFI = 0.99; TLI = 0.98; RMSR = 0.005.

#### Grit

The Duckworth and Quinn (2009) *Short Grit Scale*, made up of 8 items, validated in Spanish by Marentes-Castillo et al. (2019), was used. This instrument has two dimensions: consistency of interests (e.g., “I often set a goal, but then I follow another”) and perseverance of effort (e.g., “Setbacks do not discourage me”). The sentence that precedes these items is “In my subject …” and the responses are valued on a five-point Likert-type scale, between 1 (*Strongly disagree*) and 5 (*Strongly agree*). The results of confirmatory factor analysis were satisfactory: *χ*^2^ = 23.32; *p* = 0.00; *χ*^2^/d.f. = 3.89; CFI = 0.90; NFI = 0.92; TLI = 0.91; RMSR = 0.05.

#### Basic Psychological Needs

The Spanish version of the *Échelle de Satisfaction des Besoins Psychologiques* in the educational context (León et al., 2011) of Gillet et al. (2008) was used. The scale was preceded by the statement “In my class …” and composed of 15 items referring to academic competence (e.g., “I have the feeling of doing things well”), to academic autonomy (e.g., “I generally feel free to express my opinions”), and to the relationship with other academics (e.g., “I feel good with the people with whom I interact”). Responses were established on a Likert-type scale ranging from 1 (*Strongly disagree*) and 5 (*Strongly agree*). The results of confirmatory factor analysis were satisfactory: *χ*^2^ = 94.12; *p* = 0.00; *χ*^2^/d.f. = 3.56; CFI = 0.90; NFI = 0.90; TLI = 0.91; RMSR = 0.06.

#### Intrinsic Motivation

To measure student motivation, the intrinsic motivation to achievement subscale of the translated and validated version of Núñez et al. (2005) from the Échelle de Motivation en Éducation (EME; Vallerand et al., 1989) was used. The dimension is made up of four items (e.g., “For the satisfaction I feel when I excel in my studies”). It is preceded by the phrase “Why do you study this subject?” and the responses are collected on a Likert scale ranging from 1 (*Strongly disagree*) to 7 (*Strongly agree*). The results of confirmatory factor analysis were satisfactory: *χ*^2^ = 21.12; *p* = 0.34; *χ*^2^/d.f. = 2.10; CFI = 0.96; NFI = 0.96; TLI = 0.97; RMSR = 0.05.

#### Group Cohesion

To assess group cohesion, the group cohesion scale of Chin et al. (1999) was used. It is made up of 6 items (e.g., “I feel like I belong to this group”) preceded by the phrase “In this subject, when I work in small groups …” The results of confirmatory factor analysis were satisfactory: *χ*^2^ = 43.09; *p* = 0.06; *χ*^2^/d.f. = 3.92; CFI = 0.94; NFI = 0.95; TLI = 0.93; RMSR = 0.02.

#### Satisfaction With Life

The *Life Satisfaction Scale* (ESDV-5) of Vallerand et al. (1989), validated in Spanish by Atienza et al. (2000, 2003) was used. It consists of five items to assess the life satisfaction factor (e.g., “I am satisfied with my life”). The previous sentence is “Satisfaction with your life…” and the responses are collected on a Likert-type scale that ranges from 1 (*Strongly disagree*) to 7 (*Strongly agree*). The results of confirmatory factor analysis were satisfactory: *χ*^2^ = 33.61; *p* = 0.12; *χ*^2^/d.f. = 2.810; CFI = 0.97; NFI = 0.98; TLI = 0.96; RMSR = 0.03.

### Process

The research was approved by the Academic Council and the Board of Directors within the framework of the CONV-14-2019 Call and was approved with the code INV.140-01-007-14 at the Universidad de la Costa (Colombia). After previously establishing contact with the direction of the Academic Department, the teachers involved were contacted to inform them of the research objective and request their collaboration so that the students could fill in the questionnaires during their class time. To ensure a greater number of participants, the questionnaires were administered during their regularly scheduled classes. The application was not made in the same subject, since none is repeated throughout the different semesters of the study plan. The objective of the study and how to fill in the questionnaires was explained to the students, answering any questions that could have come up during the process. In a particular way, the students were instructed to answer the questionnaires, not bearing in mind a specific subject, but rather their general experience in relation to the development of those they have taken throughout their university education. Although initially the sample consisted of 521 students, responses with outliers were presented in 32 subjects and it was decided to eliminate them. The willingness to participate and anonymity were emphasized so that the students could feel free to answer with honesty and sincerity. The time required for its completion was approximately 20 min.

### Analysis of Data

Structural Equation Models (SEM) is a multivariate statistical technique for testing and estimating causal relationships from statistical data and qualitative assumptions about causality. First, descriptive statistical analyzes (mean and standard deviations) were performed, the internal consistency of each factor was calculated using the Cronbach’s alpha coefficient and the bivariate correlations of all the variables under study. To check the relationship between the variables proposed in the study, the two-step method was used, as it allows testing complex relationships between variables (observed and latent) with multiple ways. The first component or step is the measurement model, focused on the relationships between theoretical constructs and their observed indicator variables, in order to attribute the unobservable latent variables of multiple observed indicator variables. These possible (hypothetical) relationships are examined in the structural model or structural equations (second component) depending on the theoretical frameworks. The estimates of the parameters are free from the incidence of measurement errors because these are taken into account in the measurement model (Wang et al., 2017). In the first step (measurement model) a confirmatory factor analysis (CFA) was performed. This analysis allowed confirming the factorial structure of the scales used in the study, as well as testing their construct validity. To carry out the analysis of the measurement model and test the structural equation model, the number of latent variables of each of the factors that measured the different scales used was reduced, since it is advisable when the sample size is not large in comparison with the number of variables in the model (Marsh et al., 1994; Vallerand, 1997). This reduction can be done by combining the items in pairs. In this way, half of the first items of each subscale were averaged to form the first block of items and the second half was averaged to form the second block of items, and so on down to the last factor. Once the items that make up the latent factors were divided into two random groups, a confirmatory factor analysis was performed, based on 13 observed measures (two for each of five latent constructs and three for that of the BPN and the six latent constructs that freely correlated).

The maximum likelihood estimation method and the covariance matrix between the items were used as input for the data analysis. Similarly, the contribution of each of the factors to the prediction of other variables was examined using standardized regression weights. In the second step, the structural equation model allowed to test theoretical models including all variables within the same regression model, taking more than one dependent variable, as well as considering the same variable as both dependent and independent (Klem, 1995). The model also made it possible to discover relationships that can be incorporated or suppressed for a better fit, through modification indices, which in order to be accepted met the conditions of sensibly improving the level of fit of the model and being able to theoretically justify the proposed changes (Cea, 2002). In this way, it was proposed to measure the predictive power of support for teacher autonomy, grit, basic psychological needs, intrinsic motivation on group cohesion, and satisfaction with life. A structural equation modeling procedure to test hypothesized model was conducted. The model adequacy was assessed according to the following goodness-of-fit indexes: Comparative Fit Index (CFI), Tucker-Lewis Index (TLI), and the Root Mean Square Error of Approximation (RMSEA) with its respective Confidence Interval (CI90%). For cutoffs, CFI and TLI ≥ 0.90, and RMSEA ≤0.80 were considered as acceptable. The Confidence Interval at 95% (CI95%) was considered to measure direct and indirect effect among constructs, accepting significance if the CI does not encompass zero. To test multi-group analysis, the structural SEM model was initially assessed in each group separately. Current research adopted differences in CFI, TLI, and RMSEA to evaluate structural invariance. Structural invariance was considered to be acceptable when differences were ≤0.010 (Cheung and Rensvold, 2002). The data was analyzed using the statistical packages SPSS 25.0 and AMOS 24.

## Results

### Descriptive and Correlation Analysis of All Variables

Autonomy support presented an average value of 4.11 out of 5. In the subfactors of the grit scale, consistency presented a higher mean than perseverance. Among the basic psychological needs, the mean was higher in the perceived competence sub-factor, followed by the relationship with others and autonomy. Intrinsic motivation presented a value of 6.08, group cohesion of 5.62, and satisfaction with life of 5.72. Table 1 shows how the variables correlated positively and significantly with each other, except for perseverance with group cohesion. Regarding internal consistency, for Autonomy Support, Cronbach’s alpha values of 0.86 were obtained. For grit, values of 0.73 were obtained for the subscale of persistence of interests and of 0.80 for the subscale of perseverance of effort. For Basic Psychological Needs, internal consistency was 0.88 for competence, 0.84 for autonomy, and 0.87 for relationship with others, and jointly 0.93. For intrinsic motivation, a Cronbach’s alpha of 0.79 was obtained. For group cohesion, a value of 0.95 was obtained. Finally, for satisfaction with life, a Cronbach’s alpha of 0.90 was obtained.

**Table 1:** Mean, standard deviation, and correlations between variables.

	*M*	*SD*	*α*	1	2	3	4	5	6	7	8	9
1. Autonomy support	4.11	0.59	0.86	-	0.31^**^	0.15^**^	0.22^**^	0.33^**^	0.34^**^	0.33^**^	0.22^**^	0.22^**^
2. Consistency	4.04	0.75	0.73	-	-	0.30^**^	0.31^**^	0.32^**^	0.29^**^	0.25^**^	0.26^**^	0.35^**^
3. Perseverance	3.45	1.00	0.80	-	-	-	0.10^**^	0.14^**^	0.16^**^	0.11^**^	0.07	0.14^**^
4. Autonomy	4.05	0.77	0.78	-	-	-	-	0.62^**^	0.55^**^	0.20^**^	0.35^**^	0.40^**^
5. Relationship with others	4.31	0.66	0.85	-	-	-	-	-	0.69^**^	0.36^**^	0.56^**^	0.47^**^
6. Competence	4.46	0.57	0.82	-	-	-	-	-	-	0.35^**^	0.36^**^	0.38^**^
7. Intrinsic motivation	6.08	1.04	0.79	-	-	-	-	-	-	-	0.41^**^	0.38^**^
8. Group cohesion	5.62	1.30	0.95	-	-	-	-	-	-	-	-	0.54^**^
9. Satisfaction with life	5.72	1.18	0.87	-	-	-	-	-	-	-	-	-

** p < 0.01.

### Measurement Model

To analyze the relationships and interactions between the variables of the model that is proposed (autonomy support, consistency and perseverance, basic psychological needs, intrinsic motivation, group cohesion and satisfaction with life), the structural equation model was used. A series of indices were taken into account [*χ*^2^, *χ*^2^/d.f. = l, CFI (comparative fit index), NFI (normed fit index), TLI (Tucker Lewis index) and RMSEA (root mean square error of approximation)]. All the variables showed skewness and kurtosis values of <|2| and <|7|, respectively. On the other hand, Mardia’s multivariate index was found above 70, so it can be inferred that there was no multivariate normality (Rodríguez and Ruiz, 2008). The maximum likelihood estimation method and the covariance matrix between the items were used as input for data analysis. The indices obtained after the analysis were *χ*^2^ = 260.79; *p* = 0.00; *χ*^2^/d.f. = 4.49; NFI = 0.90; CFI = 0.92; TLI = 0.90; RMSEA = 0.08. These data adjust to the established parameters, so the proposed model can be accepted as good (Hu and Bentler, 1999). Similarly, the contribution of each of the factors to the prediction of other variables was examined using standardized regression weights. These weights range from 0.48 to 0.81. The t value associated with each weight was taken as a measure of contribution, so that values greater than 1.96 are considered significant.

### Structural Regression Model

**Figure 1:**
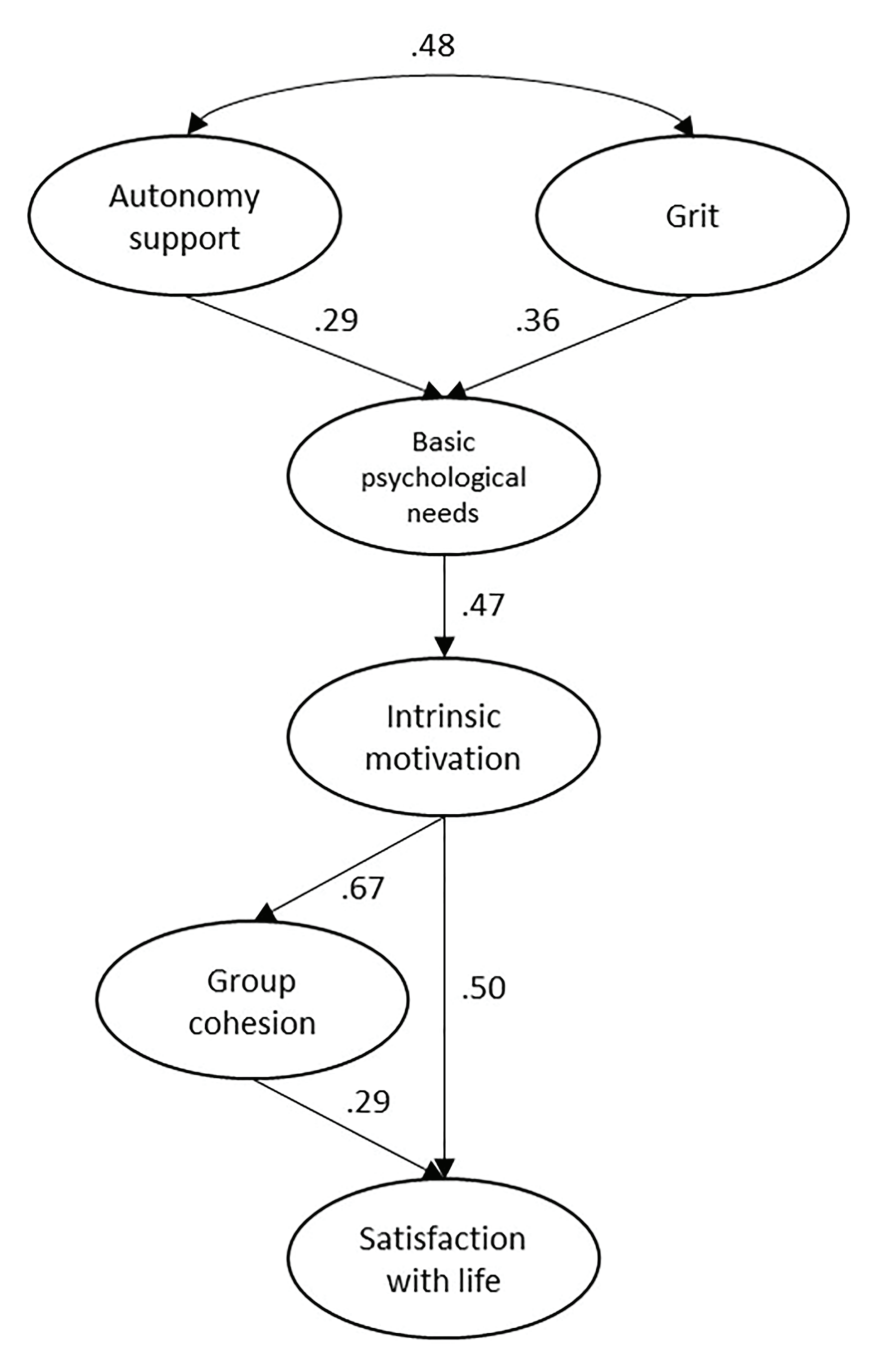
Structural equation model. The parameters are significant at *p* < 0.05 and standardized.

The indices obtained after the analysis presented an adequate adjustment model (Figure 1): *χ*^2^ = 124.56; *p* = 0.00; *χ*^2^/d.f. = 2.49; NFI = 0.90; CFI = 0.95; TLI = 0.95; RMSEA = 0.05.

### Analysis of Measurement Invariance by Sex and Age Groups

In the analysis of invariance across sex, the objective was to establish whether the structure of the confirmatory factor analysis was invariant in two independent subsamples, one of men and the other of women, by means of a multigroup analysis. The results as shown in Tables 2 and 3 showed that the four models compared had good fit indices. The differences found between the unrestricted model (model 1) and the model with invariance in factorial weights (model 2) were not significant (*χ*^2^ = 14.05, *df* = 7, *p* = 0.10). Regarding age, the entire sample was grouped into two groups (18–20 years and +20 years), after the analysis, the differences found between the model without restrictions (model 1) and the model with invariance in the weights factorials (model 2) were not significant (*χ*^2^ = 8.5705, *df* = 6, *p* = 0.10). This allows establishing a minimum acceptable criterion to consider the existence of invariance in the measurement model with respect to sex and age groups (Byrne et al., 1989; Marsh, 1993).

**Table 2:** Multigroup analysis of invariance of the model by sex.

Models	*χ*^2^	*g.l.*	*χ*^2^/*g.l.*	Δ*χ*^2^	Δ*g.l.*	CFI	IFI	RMSEA
Model 1	31.17	18	1.73	-	-	0.91	0.91	0.06 [0.056, 0.072]
Model 2	14.05	7	2.01	8.14	5	0.91	0.91	0.06 [0.056, 0.071]
Model 3	10.51	6	1.75	23.23	6	0.91	0.91	0.06 [0.055, 0.070]
Model 4	55.64	16	3.47	32.85^*^	12	0.90	0.90	0.06 [0.056, 0.072]

Model 1 = no restrictions; Model 2 = invariant measurement weights; Model 3 = invariant structural covariances; Model 4 = invariant measurement residuals.

* *p* < 0.05.

**Table 3:** Multigroup analysis of invariance of the model by age.

Models	*χ*^2^	*g.l.*	*χ*^2^/*g.l.*	Δ*χ*^2^	Δ*g.l.*	CFI	IFI	RMSEA [90% CI]
Model 1	23.32	18	1.29	-	-	0.91	0.91	0.06 [0.055, 0.071]
Model 2	8.57	6	1.22	9.43	5	0.91	0.91	0.06 [0.055, 0.070]
Model 3	14.63	9	1.62	17.76	6	0.91	0.91	0.06 [0.054, 0.072]
Model 4	42.42	13	3.26	28.15^*^	9	0.90	0.90	0.06 [0.056, 0.072]

Model 1 = no restrictions; Model 2 = invariant measurement weights; Model 3 = invariant structural covariances; Model 4 = invariant measurement residuals.

* *p* < 0.05.

## Discussion

This study tested a model that emphasized the predictive capacity of a high perception of teacher’s autonomy support and student grit to improve life satisfaction in university students, being mediated by the satisfaction of basic psychological needs, intrinsic motivation, and group cohesion. The results confirmed the hypothesis. Furthermore, all variables were positively and significantly correlated with each other, except perseverance with group cohesion. It is confirmed that the interpersonal style of autonomy support, as well as the grit, both as triggers in the motivational process, positively predict basic psychological needs and intrinsic motivation, and the latter predicts group cohesion and satisfaction with life.

Of the three basic psychological needs, it is the relationship with others that presented the greatest correlation with intrinsic motivation, which is consistent with the fact that, in turn, the relationship with others correlated significantly with group cohesion. This highlights the importance of the relationships within the groups for life satisfaction. Corroborating this statement from previous research, Datu (2017) points out that the sense of relationship with others (teachers and parents) is linked to a higher value in societies where proximity in relationship prevails over individualism, and it is associated with greater consistency and perseverance.

In general, these results also coincided with other studies (Moreno-Murcia and Silveira, 2015) in which they found that students with greater self-determination developed deep study processes and were more satisfied with life. In this same sense, Clark and Malecki (2019) found consistent and positive associations between academic determination and academic performance, life satisfaction and school satisfaction, although in a group of high school adolescents. Along the same line, other investigations have shown that intrinsic motivation is related to greater learning, as well as greater permanence in the training process and achievement (Depasque and Tricomi, 2015; Griffin, 2016; Leenknecht et al., 2017; Orsini et al., 2018).

The evidence obtained from this research places grit as a social trigger in the motivational model. It is striking that both dimensions, consistency and perseverance, also predict basic psychological needs and intrinsic motivation, as this predictive relationship is usually related to the teacher’s interpersonal style. In this same direction, Ka and Zhoun (2019) and Tynan et al. (2020) also place them at the same level as a predictor of academic success.

Similarly, additional evidence from this study showed that group cohesion mediates with satisfaction with life, in the same way that Robbins and Madrigal (2019) in relation to performance and well-being. Also, Ambrey et al. (2017) highlight the importance of social connections in relation to social well-being. Likewise Beattie et al. (2018) and Farruggia et al. (2018) conclude in their studies on non-cognitive factors associated with academic success in university students, that the academic mentality, in relation to the sense of belonging to a reference group, is related to academic success.

Therefore, the results of this study highlight SDT’s postulates regarding the importance of taking into account both contextual and personal factors in the educational field to promote positive results. In this sense, we think that the teacher could take into account that this will be possible to achieve when interaction with their students is perceived with high autonomy support, but also when consistency and perseverance are high. Our recommendation, based on the evidence from this work: it would be advisable for the teacher to focus, especially within their style of autonomy support, on those strategies that foster a committed interest in the task along with the teacher’s sustained accompaniment over time and always focused on a realistic goal. With this and given the existing correlation with group cohesion that is fueled by the psychological need for a relationship with others in which the student feels a connection with others, the teacher will be able to contribute to increasing the well-being of the student.

The present study contributes to the literature insofar as it assesses the mediating effect of screaming, in relation to autonomy support, BPN, group cohesion, and satisfaction with life. The study confirmed previous findings in the sense that teachers have a decisive influence on satisfaction of BPN, intrinsic motivation, and satisfaction with life, and thus, highlights the need to create student-friendly climates. But also, in a similar way, it showed that grit also plays an important role in this process and, therefore, the urgency for teachers to become facilitators to enhance in their students a sense of consistency and perseverance, as well as a greater sense of group cohesion in their active participation in learning scenarios.

One of the limitations of the study is that, having a correlational scope, only correlations are established between the variables treated, and although the structural equation model allows a prediction to be made, it is not possible to establish a causal relationship. Experimental studies that explain the causal relationships of the studied variables, and others in which the sample is randomized and equally distributed by gender, are necessary. In addition to the issue of scope, the type of cross-sectional design adopted does not allow an analysis to be advanced in a longer timeline. This makes it necessary for subsequent studies to measure the evolution of the variables in various temporal cuts. Furthermore, the proposed model is the one that presented the best fit, but due to the problem of equivalent models presented by the technique of structural equations (Hershberger, 2006), it is assumed that the proposed model would be only one of the possible ones. Another limitation is that the study was developed from a brilliant motivational process model and did not take into account the dark path posed by the dual process, thus it could not have considered other possible explanations around the impact of both social and personal factors in relation to with satisfaction with life. A final limitation has to do with the selected sample, since it was only about university students. Future studies may consider other educational levels such as primary or secondary education.

In conclusion, both the interpersonal style of autonomy support and the grit, as well as the establishment of solid interpersonal relationships, are key factors associated with the satisfaction of basic psychological needs, motivation, and well-being. As practical implications, in a higher education setting, the consideration of certain personal student variables related to self-regulation should be elements that serve as a basis to complement and guide effective pedagogical practices based on promoting autonomy support and strengthening the processes of permanence and success of students. From this, teachers have the opportunity to enhance student motivation through pedagogical strategies that promote group cohesion (Bronson, 2016). This represents a challenge, since according to Ryan and Deci (2020) conventional relationship styles are installed under the protection of institutional models and educational policies conventionally centered on control practices.

## Data Availability Statement

The raw data supporting the conclusions of this article will be made available by the authors, without undue reservation.

## Ethics Statement

The studies involving human participants were reviewed and approved by the Academic Council and the Board of Directors within the framework of the CONV-14-2019 Call, and were approved with the code INV.140-01-007-14 at the Universidad de la Costa (Colombia). The patients/participants provided their written informed consent to participate in this study.

## Author Contributions

JL-J advanced the relationship process with the University to have the guarantees to carry out the study, as well as the contact with the professors and the management of the informed consent of the students. In that same sense, the administration process of the surveys and their initial tabulation. JM-M and EH led the data processing and analysis process, with the support of JL-J. Finally, all authors structured the final version of the manuscript after exhaustive reviews of documents relevant to the research.

### Conflict of Interest

The authors declare that the research was conducted in the absence of any commercial or financial relationships that could be construed as a potential conflict of interest.
